# Mapping the terraces on the Loess Plateau based on a deep learning-based model at 1.89 m resolution

**DOI:** 10.1038/s41597-023-02005-5

**Published:** 2023-03-02

**Authors:** Yahan Lu, Xiubin Li, Liangjie Xin, Hengfei Song, Xue Wang

**Affiliations:** 1 Key Laboratory of Land Surface Pattern and Simulation, Institute of Geographic Sciences and Natural Resources Research, Chinese Academy of Sciences, Beijing, 100101 PR China; 2 University of Chinese Academy of Sciences, Beijing, 100049 PR China

**Keywords:** Ecology, Hydrology, Environmental impact

## Abstract

Terraces on the Loess Plateau play essential roles in soil conservation, as well as agricultural productivity in this region. However, due to the unavailability of high-resolution (<10 m) maps of terrace distribution for this area, current research on these terraces is limited to specific regions. We developed a deep learning-based terrace extraction model (DLTEM) using texture features of the terraces, which have not previously been applied regionally. The model utilizes the UNet++ deep learning network as its framework, with high-resolution satellite images, a digital elevation model, and GlobeLand30 as the interpreted data and topography and vegetation correction data sources, respectively, and incorporates manual correction to produce a 1.89 m spatial resolution terrace distribution map for the Loess Plateau (TDMLP). The accuracy of the TDMLP was evaluated using 11,420 test samples and 815 field validation points, yielding classification results of 98.39% and 96.93%, respectively. The TDMLP provides an important basis for further research on the economic and ecological value of terraces, facilitating the sustainable development of the Loess Plateau.

## Background & Summary

Terraces have become a major source of arable land and a soil erosion control measure on the Loess Plateau^1,2^. The Loess Plateau is the world’s largest loess sedimentary deposit. Due to its loose soil texture, combined with the impacts of climate change and past irrational land use, it has been one of the most susceptible areas to soil erosion in the world^3^. As the Chinese government has promoted soil erosion control measures in the region over the last few decades, the proportion of eroded soil has decreased from 72.76% to 36.1%^4^, a reduction of 237,800 square kilometres. As a result, the Yellow River, the world’s largest sand-bearing river, has also become “clearer”^5^. Terracing projects are widely regarded as one of the most important soil erosion prevention measures because of their economic and ecological benefits^6–9^. However, due to the lack of a high-resolution (<10 m) terrace distribution map for the Loess Plateau (TDMLP), current research has been limited to specific areas^10^, and research on the Loess Plateau has not yet been studied comprehensively. The absence of sufficient spatial detail may create uncertainty in the assessment of terracing-related studies; therefore, it is crucial to develop a high-precision and high spatial resolution TDMLP to study sustainable development in the region.

Terraces are difficult to extract using traditional classification methods due to their multi-modality and complexity. Traditional classification methods fall into three main categories: some use pixel-based classification combined with state-of-the-art automatic classification algorithms such as random forests^11–14^; others employ object-oriented classification combined with semi-automatic classification algorithms such as decision trees^15^, and still others use visual interpretation^16,17^. On the one hand, the first methods will rely more on spectral characteristics for classification, making it difficult to distinguish between sloping land and terraces. Terraces are defined based on their morphological features; their spectral characteristics are poor. On the other hand, the resolution of 30 m is insufficient for detailed and in-depth terracing studies, including terrace abandonment studies^18^. Because of topographic restrictions, patches of terrace are typically small and irregular in size, resulting in mixed image elements^19^ and pretzel phenomena^20^, which are common and affect product accuracy. Currently, high-resolution satellite imagery can be used to overcome these shortcomings^21^. Although high-resolution satellite images (RGB) lack spectral information, they contain a wealth of information regarding the colour, morphology, texture, and structure of different features. The breadth of coverage is also ideal for classified terraces. While the second methods can utilise rich textural and spectral features to classify terraces, current classification in the Loess Plateau is restricted to specific areas due to the multimodality and complexity of terraces in this region. Despite being extremely accurate, manual visual interpretation has rarely been performed on the Loess Plateau due to its high economic and time costs.

Deep learning is a powerful machine learning algorithm that is widely used in computer vision and natural language processing^22,23^. It could be a viable option for helping humans to manage big data. By simulating human visual mechanisms, the development of deep learning in computer vision aids semantic segmentation of images after fully considering the morphological texture characteristics of objects. Deep learning has been developed and is currently fully applied in computer vision^24–26^. Researchers are now combining deep learning with remote sensing images for geo-extraction applications^27^. The UNet++ network^28^ is an important deep learning neural network that is widely used because it is suitable for classifying images with simple semantics and fixed structure. Since terraces are characterized based on their strong morphological structural features, simple semantics, and fixed structure, deep learning techniques are appropriate and novel methods for extracting them.

In this study, we constructed a deep learning-based terrace extraction model (DLTEM) using UNet++ as the network framework. High-resolution satellite images, a Digital Elevation Model (DEM), and GlobeLand30 were used as the interpreted data and topography and vegetation correction data sources, respectively. A TDMLP with a spatial resolution of 1.89 m was created based on the supervisory classification method combined with manual correction. In the context of socio-economic development and global climate change, this map lays the foundation for further studies on these terraces, such as those on food security and ecological conservation in mountainous areas.

## Methods

**Fig. 1:**
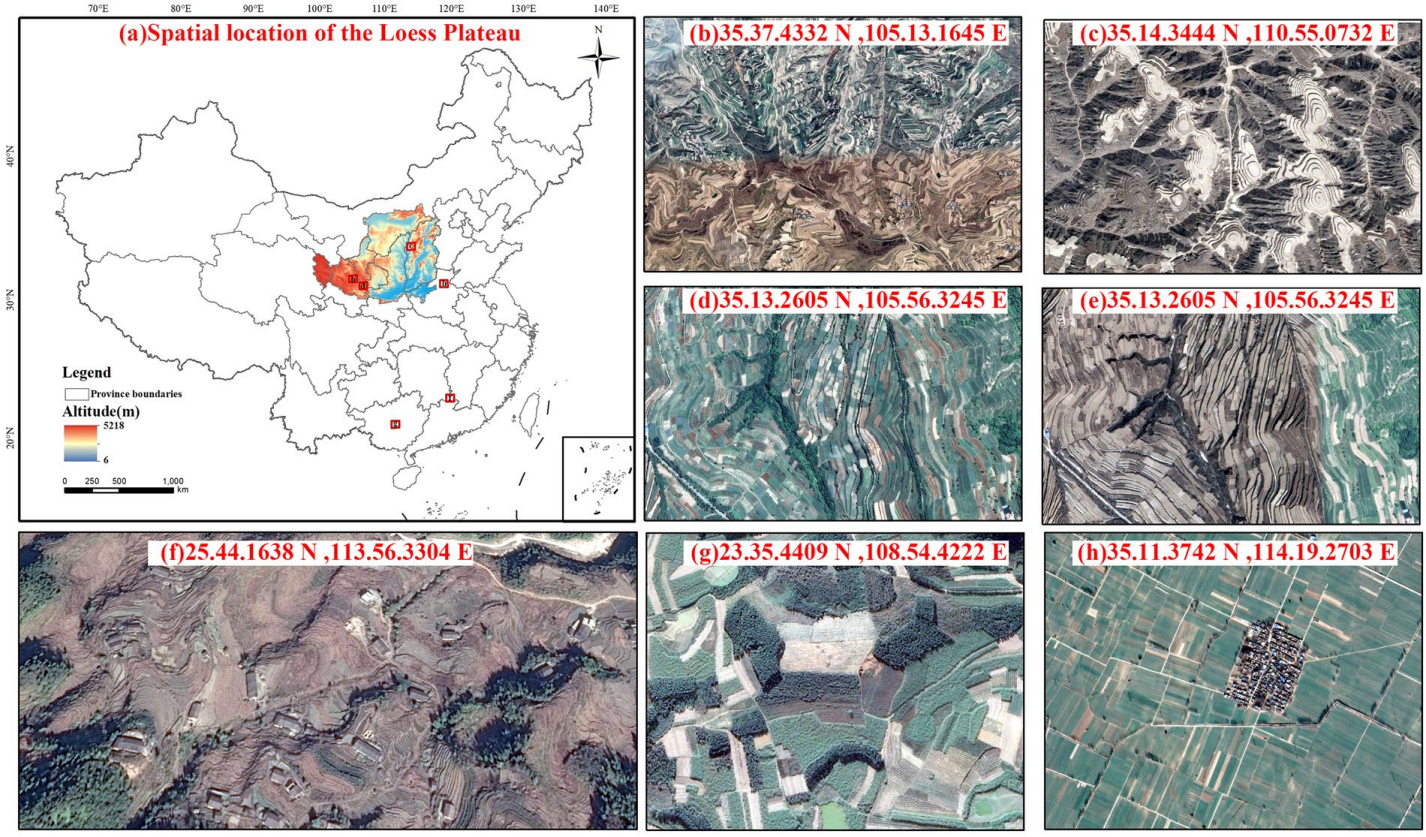
The spatial location of the Loess Plateau and images of various types of cultivated land. (**a**) The spatial location of the Loess Plateau and Spatial distribution of various cultivated land types images, (**b**) wide strip-mounted terraces in Longxi, (**c**) circular wide terraces in central Yulin, (**d**) high resolution image of Zhuanglang County in July 2019, (**e**) Zhuanglang County in February 2020, (**f**) narrow terraces in Shangbao, Chongyi, Jiangxi Province, (**g**) sloping cropland in Zhenjiang Town, Laibin, Guangxi, and (**h**) horizontal cropland in the North China Plain.

Terraces are a land type that is defined by its shape. They have a distinct morphological structure and edge features that distinguish them from other land types. In this study, we define terraces as agricultural land with strip or wavy sections built on slopes greater than 2° along the contour direction. Figure 1 depicts Google Maps satellite images of terraces in the Loess Plateau region. Terraces can be distinguished from other features in remote sensing images based on their colour, morphology, texture, and structure. Terraces can be distinguished from construction land, water, glaciers, and deserts by their colours. Figure 1b–d shows terraces that are primarily green and yellow. Furthermore, terraces are generally distributed along the contour direction, and can therefore be identified based on their morphology. Terraced field ridges curve downward and resemble strips in Fig. 1b,d or circles or ovals in Fig. 1c rather than a neat grid-like distribution. These features differ in morphology from the flat land shown in Fig. 1h. Based on texture and structure, the field area of terraces can be identified based on their strong edge features, as shown in Fig. 1b–d. The edges of terraces have dark stripes caused by oblique illumination received from the sun, and the field ridge of terraces often intercepts part of the sunlight due to their height. Sloping cultivated land, as shown in Fig. 1g, has no evident terraced wall. The outline of sloping cultivated land in the high-resolution image is curved, with no prominent edge features. These findings are critical differences distinguishing terraces and sloping land in high-resolution images.

### Deep learning-based terrace extraction model

**Fig. 2:**
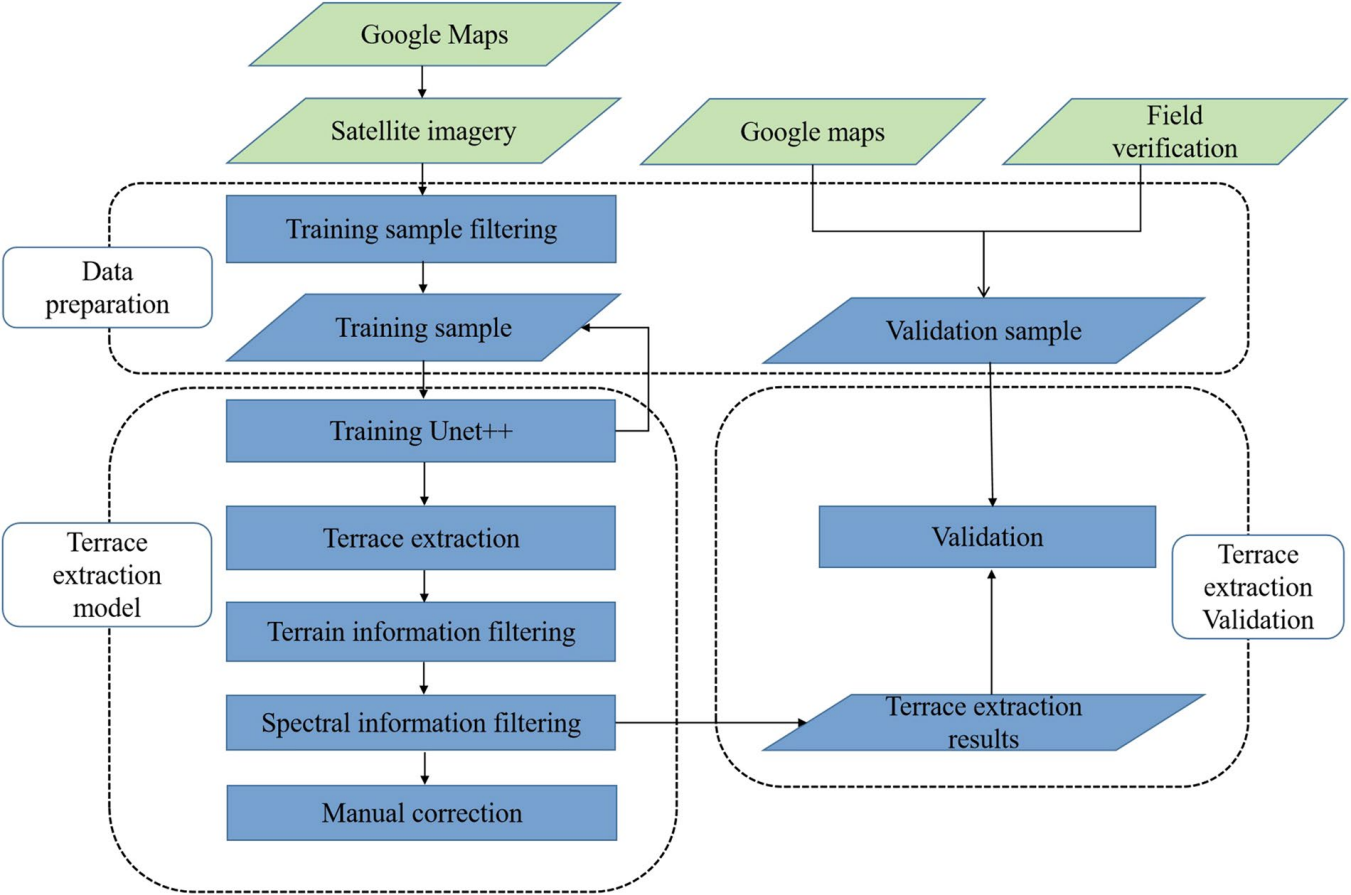
Flow chart of the deep learning-based terrace extraction model.

The DLTEM is a terrace extraction model that uses deep learning algorithms and other supplementary information. Initially, a preliminary terrace distribution map was obtained using a deep learning algorithm. It was then combined with the spectral and digital elevation model (DEM) elevation information to fine-tune the results. The final spatial distribution of the terraces was produced by manual correction (Fig. 2). Traditional land classification models or methods typically superimpose spectral, elevation, and morphological texture information from remote sensing images together for training, such as random forest, which is easily ignored in training since morphological texture information accounts for a relatively small amount of the total information. This leads to significant errors while identifying land classes with textural characteristics. In contrast, the DLTEM focuses on morphological texture information from remote sensing images and classifies it into land classes, followed by auxiliary correction through additional information. Thus, this method is more suitable to extract terraces enriched with texture structure information.

The UNet++ network is a classic deep learning algorithm that is uniquely unrivaled in extracting colour, morphology, texture, and structure features from images and applying them for classification. In comparison with other Convolutional Neural Network (CNN) classification models (e.g., Fully Convolutional Networks (FCN)), it has high classification accuracy, fast computation speed, strong robustness, and provides variable importance metrics. Therefore, in this study, the UNet++ network was adopted as the network framework for deep learning; the primary data source used was high-resolution satellite imagery from 2019. DEM (SRTM v4.1) data were used to obtain the elevation information and GlobeLand30 data were used to obtain the spectral information. The results were corrected to construct the final map of the distribution of terraces in the Loess Plateau.

### Study area

The Loess Plateau, one of China’s four major plateaus, is located in northern central China (34°–40° N and 103°–114° E) (Fig. 1). It is covered by a thick loess layer that ranges in thickness from 50 to 80 m, and is the world’s largest loess deposition area, covering 648,700 km^2^. The altitude of the Loess Plateau ranges from 800 to 3,000 m, its average annual temperature is 6–14 °C, and its average annual precipitation is 200–700 mm. Since ancient times, the Loess Plateau has been used for agriculture because of its fine grains, fluffy soil texture, and rich soluble mineral nutrients, all of which are conducive to crop cultivation. However, long-term unsustainable land use caused the degradation of the vegetation cover in the Loess Plateau. Moreover, the land is degrading due to considerable nutrient loss caused by long-term water erosion in conjunction with natural conditions, such as arid climate, loose soil, concentrated and heavy rainfall. The fragmented ground in the region has made it susceptible to soil erosion. It has also become the primary source of Yellow River sediment as a result of the massive flow of eroded sediment into the Yellow River, posing a serious threat to the economic and social development of the lower Yellow River basin.

Terracing is one of the main measures used to enhance crop yield and conserve soil and water in the region. Since the 1980s, the Chinese government has implemented many large-scale slope-to-terrace projects in the Loess Plateau. Especially in recent years, the outline of the comprehensive management plan for the Loess Plateau area (2010–2030) has been promulgated with a planned area of 2.608 million hectares for slope to terrace conversion, making it the core area of slope to terrace conversion projects in the country.

### Data preparation

Although high-resolution satellite images can be an important data source for the spatial distribution of terraces on the Loess Plateau, they are not ideal for terraces classification. On the one hand, a higher resolution image requires more storage space. On the other hand, it reduces the efficiency, prolongs the interpretation time, and increases the noise in the image, affecting the interpretation accuracy. Most of the terraces on the Loess Plateau are wider than 7 m (Fig. 1b–d). These are wide terraces in comparison with the narrow terraces of southern China (Fig. 1f), which are less than 2 m wide. Furthermore, it is also easy to mistake the fish-scale pits constructed for soil and water conservation for terraces because of their similarity in form. However, as the width of their field surface is less than 1.5 m, remote sensing images with a 2 m resolution can effectively prevent the false extraction of such features. Based on the actual situation of this study area, we chose a high-resolution image with a spatial resolution of 1.89 m from Google Maps 16 level as the data source. The colour, texture, and morphological features of terraces in the images show seasonal variations. In autumn and winter, the weather is dry, and the vegetation is less shaded in the Loess Plateau. During this time, even the edge features become more visible and easier to identify. As a result, we selected images from October 2018 to February 2019 whenever possible (Fig. 1c,d).

### Deep learning network selection

Land classification is the extraction of land types from remote sensing images using image segmentation techniques. As the key technology of image segmentation, the Fully Convolutional Network (FCN) classifies images at the pixel level. FCN follows the network structure pattern of encoding and decoding, which adopts AlexNet as the encoder of the network and then employs transposed convolution to up-sample the feature map output from the final convolutional layer of the encoder to the resolution of the input image to achieve pixel-level image segmentation. However, due to the large error in image pixel boundary localization, Ronneberger *et al*.^29^ improved the FCN structure in 2015 by expanding the capacity of the network decoder by adding a contracting path to the encoding and decoding modules to achieve more accurate pixel boundary localisation^29^. The U-Net network is commonly used in medical image processing because it requires a small number of training samples and is effective in classifying objects with a fixed structure and limited semantic information. This network is comparable to natural image semantic segmentation such as Deeplab v3+, which has a smaller number of model parameters and the same effect.

Since the texture and morphological features of terraces and human organs have certain similarities, they are primarily manifested by simple semantic information contained within the terrace images themselves. Thus, high-level semantic information and low-level features of such images become more important. However, high-resolution images are more complicated and variable than medical image patterns, and errors in terrace extraction edge identification using the U-Net network, such as boundary segmentation of terraces and flatlands, still occur. To fully utilize the semantic information of the network, we adopted a nested U-Net architecture, namely the UNet++ network proposed by Zhou *et al*.^28^. The network integrates long-connected and short-connected architectures to capture features at different levels by adding a shallower U-Net structure and integrates them via feature superposition to make the scale difference of feature maps smaller when fused to enhance the correct rate of image segmentation edges. However, because the U-Net++ network increases the number of model parameters, this study adopted the sparse matrix approach to accelerate model training and decrease the number of parameters.

### Data pre-processing

Data pre-processing is a prerequisite for UNet++ network training, that is, valid input according to the standard format annotation before training can be performed. Since the UNet++ network proposed by Zhou *et al*.^28^. is primarily used for medical images, which have characteristics such as fixed image structure, no spatial information, and less pattern variation, labelling medical images is comparatively easier using this method. In contrast, high-resolution remote sensing images have a large number of rasters, many pattern changes, irregular image structure, and spatial information. Therefore, determining how to better annotate high-resolution remote sensing images and reduce the annotation workload becomes critical. First, we vectorized the training sample area and generated the terrace vector dataset using ArcGIS with a high-resolution remote sensing image as the primitive map. Second, we converted the terrace vector dataset into raster data. The information of the raster had to be identical to that of the primitive map, including the size of the raster, its processing range, and its coordinate system. The output was converted to TIFF format to complete the image annotation. Since the raster size input to UNet++ network training is a fixed size, it is much smaller than the original image. To simplify the process of inputting the original image and its annotation information, we added an image import module to DLTEM, which was a sliding window of 400*400, and read the image automatically by setting the corresponding judgement conditions. Finally, the entire high-resolution image was processed automatically into the model in accordance with the established rules for training.

The goal of the data enhancement was to improve the universality and robustness of the UNet++ network training results. As mentioned above, the high-resolution images taken simultaneously often included clouds or other anomalies in some areas, as the images were stitched together using multiple sources of data fusion. This can easily form evident stitching traces (Fig. 1c,d) due to the different shooting times and image quality of various data sources, i.e., brightness, saturation, and colour contrast of the images. Thus, the model trained on the original image data has strong limitations, and in many scenes, there are notable matrix-type misclassification regions due to image differences, making extraction work challenging. Therefore, in this study, we first adjusted the brightness, grayscale, and contrast of the training data after input to enhance its colour feature recognition ability. We then altered the scaling of the image, and rotated and transformed the training image from 0° to 360° to enhance morphological feature recognition and the accuracy of the training network in terrace extraction.

### Parameter setting

The network parameter setting is the most critical hyperparameter for UNet++ network training. They are mainly divided into input image size, batch size, learning rate, number of iterations, objective function, gradient descent strategy, momentum, decay rate, and activation function. Among them, we set the image size to 400*400 pixels based on the actual situation of the terraced area, where the UNet++ network has four scaling times, and the image size must be a multiple of 16. The batch size primarily affects the convergence of the model. If the batch limit is set to one, the model is easily affected by the random perturbation phenomenon and cannot converge to find the optimal solution. Since the batch size is determined by the size of the video memory, the value of the batch is limited by equipment constraints. The model in this study used a 2080Ti video card with 11 GB of video memory, and the batch was set to 8. The learning rate, gradient descent strategy, and objective function play a role in whether the network can find the best classification model better and faster. The learning rate was set to 0.001 for the first 500 generations, with the goal of achieving fast convergence to the target region. The learning rate was then set to 0.0001 for 500–1,000 generations, and the model was fine-tuned by choosing a smaller learning rate to find the model with the highest classification accuracy. Adam was chosen for the gradient descent strategy. The momentum and adaptive learning rate were used to increase the convergence rate. The cross-entropy classification loss function was chosen as the objective function to improve the differentiation between terraced and non-terraced areas. Momentum, decay rate, and activation function were all adopted from the previous default settings of the UNet++ network.

### Data correction

In this study, we primarily used high-resolution images from Google Earth as the data source to extract the distribution of terraces on the Loess Plateau. Because this image source only contains a large amount of texture structure information and no vegetation information, it is easy to misjudge and misclassify features with the same morphological structure and edge features, such as permanent snow and ice, water bodies, bare land, and artificial surfaces. Vegetation information was generally processed based on waveband data from multispectral/hyperspectral images. It requires topographic correction, atmospheric correction, radiometric calibration, de-clouding, and other operational processes, which are extremely sophisticated^30^.

GlobeLand30 is a 30 m spatial resolution global surface coverage dataset developed by the National Geomatics Center of China. The most recent GlobeLand30 dataset (v2020) has been updated with data sources from 2017 to the present. Its extensive data sources enable effective reduction of the impacts of cloud cover, with an overall accuracy of 85.72%. The classification accuracy of permanent snow and ice, water bodies, bare land, and artificial surfaces of this dataset is as high as 75.79%, 84.70%, 81.76%, and 86.70%, respectively. Since the update time of v2020 data is similar to that of high-resolution images, it can be used as correction data for vegetation information^31^.

Since the training image data are two-dimensional planar data with no elevation or slope information (Fig. 1g), certain flat fields with visible field bumps are easily misclassified as terraces. The Space Shuttle Radar Topography Mission (SRTM v4.1) DEM has a spatial resolution of 30 m and ranges from 60° N to 56° S, completely covering the Loess Plateau^32,33^. In this study, these data were treated as terrain correction data. The amendment standard corrects the areas that have been extracted as terraces below 2° to non-terraced areas according to the requirements of the Ministry of Natural Resources of China.

The spatial resolution of our extracted terraces is 1.89 m, whereas the spatial resolution of GlobeLand30 and DEM as correction data sources is 30 m, which is difficult to meet the requirements of data processing. Hence, we up-sample the two correction data sources, and then used multi-source data fusion. First, we extracted and up-sampled the terraced areas of glaciers, rivers, and deserts from GlobeLand30 to a spatial resolution of 1.89 m. Secondly, we up-sampled the DEM to 1.89 m using spatial interpolation for its raster centre as the true value of the region and performed a slope calculation for the up-sampled DEM. Further, the spatial distribution maps of glaciers, rivers, deserts, and slope maps of the Loess Plateau with the same resolution as the spatial distribution maps of terraces were available. Finally, we superimposed these images, used the terrace range in the TDMLP as a mask, and assessed the pixels in the mask area one by one. If a pixel belonged to permanent snow and ice, a water body, bare land, or an artificial surface, or had a slope less than 2°, it was modified to the background value. Otherwise, the original value was retained.

We made artificial corrections to the data based on the extracted results for the arid areas of the Loess Plateau as well as for the flatter basins, given that these areas do not feature terraces.

### Training and validation data

For supervised classification, the selection of sample areas and sample features is crucial. The focus and core of any land classification work is representative and effective training sample selection. To obtain a better sample area selection, we considered the selection of sample areas from three perspectives, i.e., colour texture features, topographic features, and spatial distance of the training samples. First, the terraces in this study are in agricultural land, including cultivated land, woodland, grassland, and other types of land; thus, different types of land will present different texture details. At the same time, high-resolution images from Google Earth are mosaicked. Because of the different acquisition times, the same region and land type will have visible colour differences and stitching traces, which is more common in the Loess Plateau region. Therefore, these factors should be considered in the selection of training samples as much as possible to improve the generability of the model and the correct rate of its extraction. Second, the state of the terraces varies according to topographic features. Among them, gradient, direction, altitude, and climate are the most significant factors. Terraces can be categorised as shallow-slope or steep-slope terraces. Based on slope aspect, altitude, and climate characteristics, they can also be categorised as either easy to identify or hard to identify. Thus, the sample should be inclusive of these types of terraces. According to the first law of geography, terraces in different spatial locations have different morphologies. Therefore, the spatial location of the samples should also be at a certain distance.

**Fig. 3:**
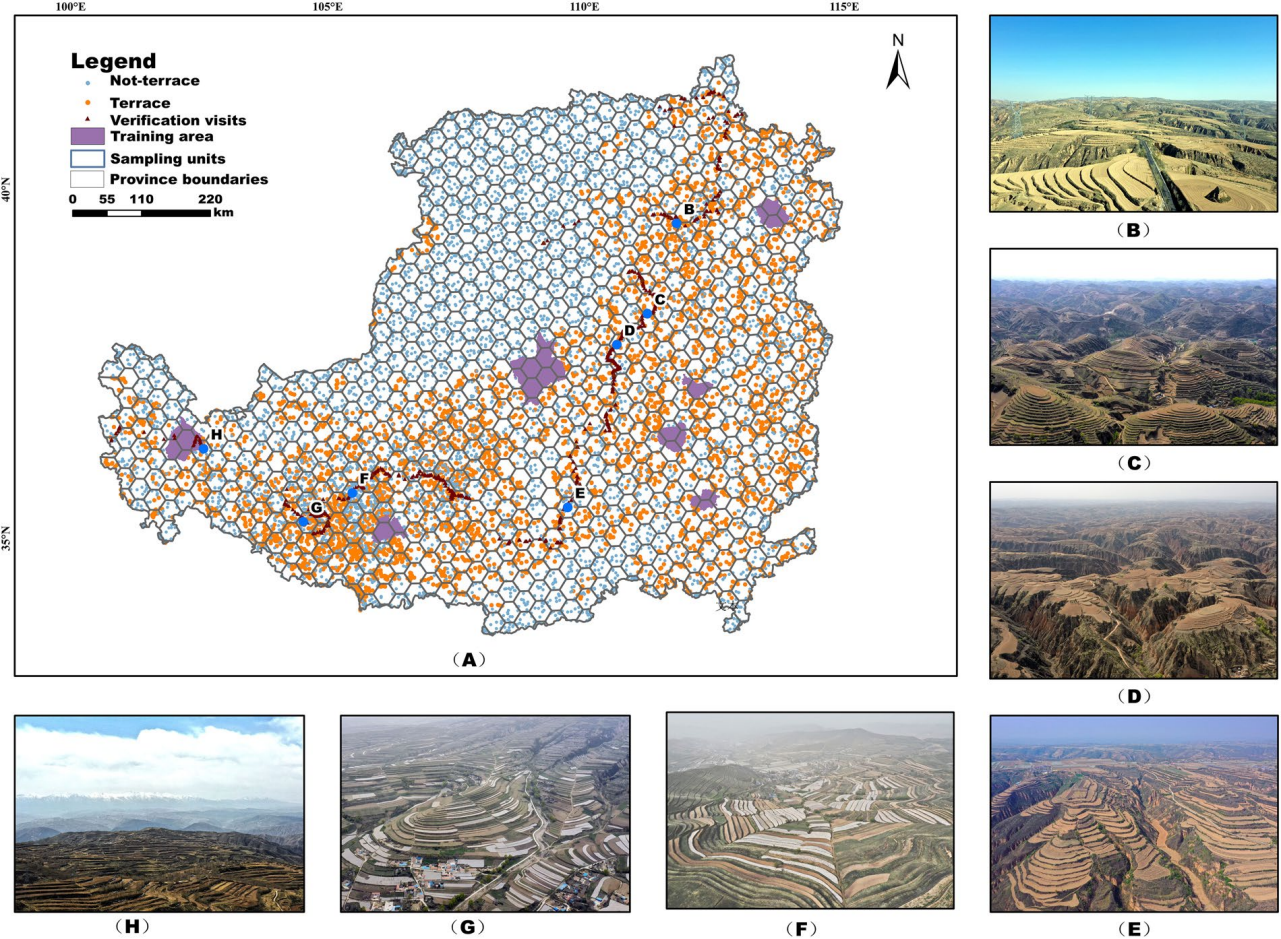
Distribution of training sample areas and validation sites in terraces on the Loess Plateau.

In summary, we selected one county in each region based on the geomorphic zoning characteristics of the Loess Plateau. In addition, we added one more in the area where the density of terraces may be higher. Finally, we selected the whole area of seven counties (Fig. 3) as the training sample area distribution, covering 2.18% of the overall Loess Plateau area. The colour morphological features, topographic features, spatial location, and imaging quality of terrace images in these regions are highly representative. This method was unique from other classification methods. Most of the traditional methods are based on the single-pixel information of feature layers such as random forests, which tend to ignore the neighbouring information around the point, and thus are subject to misclassification and under classification for land types with outstanding texture information. In our study, we adopted the visual interpretation of the whole domain, which can cover the neighbourhood information of each pixel point more comprehensively. To ensure the uniformity and correctness of visual interpretation, the terraces in the training area were visually interpreted by seven interpreters after uniform professional training. For the disputed and uncertain areas, the seven interpreters carried out interactive interpretation and scoring according to the interpretation results. Finally, two other interpretation experts made the final review and corrections. The interpretation results of the training area were re-examined and revised based on the results of the later interpretations.

To better assess and compare the validity and correctness of the terraced agricultural area datasets on the Loess Plateau in quantitatively, the validation dataset was divided into two parts: a per-pixel point-based validation set and a field validation dataset of terraces with location information. The extracted datasets were comprehensively evaluated in terms of both pixel scale and field validation.

We constructed a single-pixel validation point that evaluates the TDMLP. We applied the Icosahedral Snyder Equal Area Discrete Global Grid created by ArcGIS. Based on this strategy, the study area was partitioned into 972 regions (Fig. 3). To better validate the terrace classification results (excluding non-terrace classes), we placed more validation points within the grid where the terrace distribution is more concentrated. First, we calculated the proportion of terraces in each hexagonal grid to the total area of the hexagonal grid. Second, we separated the terraces into four levels according to the proportion of terraces to the whole grid area as 0–20%, 20–50%, 50–80%, and 80–100% and the number of validation points was 10, 20, 40, and 50, respectively.

Since the proportion of the extracted terraced area to the total area was only 14%, direct random point deployment would have led to fewer terraced validation sets and thus would have affected the final data evaluation. Therefore, in the deployment strategy, we ensured that the validation points distributed in the extracted terraces in each grid account for at least one-fifth of the total number of validation points, but for the grid with a smaller proportion of terraces or even 0, this practice was meaningless. Hence, we stipulated that in the grid with a proportion of terraces ≤1%, direct random scattering was to be performed. The final scattered verification points in the terraced and non-terraced areas were 5,194 and 6,226, respectively, with a ratio close to 1:1 for easy verification. The spatial distribution is shown in Fig. 3.

We validated the spatial distribution map of terraces on the Loess Plateau from 14 April 2021 to 1 May 2021 and constructed a field validation dataset of terraces with location information. Considering the longitudinal, latitudinal, and vertical heterogeneities of the Loess Plateau, the verification route was divided into two sections, north to south and east to west, to more comprehensively cover all regions of the Loess Plateau. The verification route started at Hohhot in the northeast of the Loess Plateau. It passed through the Datong Basin, followed the Yellow River to the south and the Weihe Plain, and then travelled westward through Mount Liupan to the westernmost part of the Loess Plateau. The route was through 54 counties/districts in 16 cities and six provinces on the Loess Plateau, with a total distance of 3,680 km, covering 15.8% of the counties on the Loess Plateau (total of 341 counties). We also surveyed and sampled the verification points approximately every 5 km along the route and collected data from a total of 815 sample points, covering various types of terraces on the Loess Plateau. The results are shown in Fig. 3.

## Data Records

The 1.89 m spatial resolution map of the proportion of terraces on the Loess Plateau is available for public use at Figshare^33^. The files are stored in GeoTIFF format with the projection coordinate system: WGS_1984_Web_Mercator_Auxiliary_Sphere. The geographic values are 0 and 1, indicating non-terraced fields and terraced fields, respectively. The TDMLP will be available after publication. The GlobeLand30 data used in this study can be downloaded from http://www.globeland30.org. The SRTM DEM data are available on the Google earth engine (GEE) platform.

## Technical Validation

Assessing the accuracy of land cover/utilisation products is a vital step in describing the reliability of the products for applications in related fields^34^. Although there is no standard method for classification accuracy evaluation, the confusion matrix is widely considered to be the best metric^35^. In this study, the confusion matrix was chosen as the evaluation method as it not only describes the confusion between various land cover types but also provides quantitative measures including user accuracy, producer accuracy, overall accuracy, and the Kappa coefficient, which can be good measures of product performance.

**Fig. 4:**
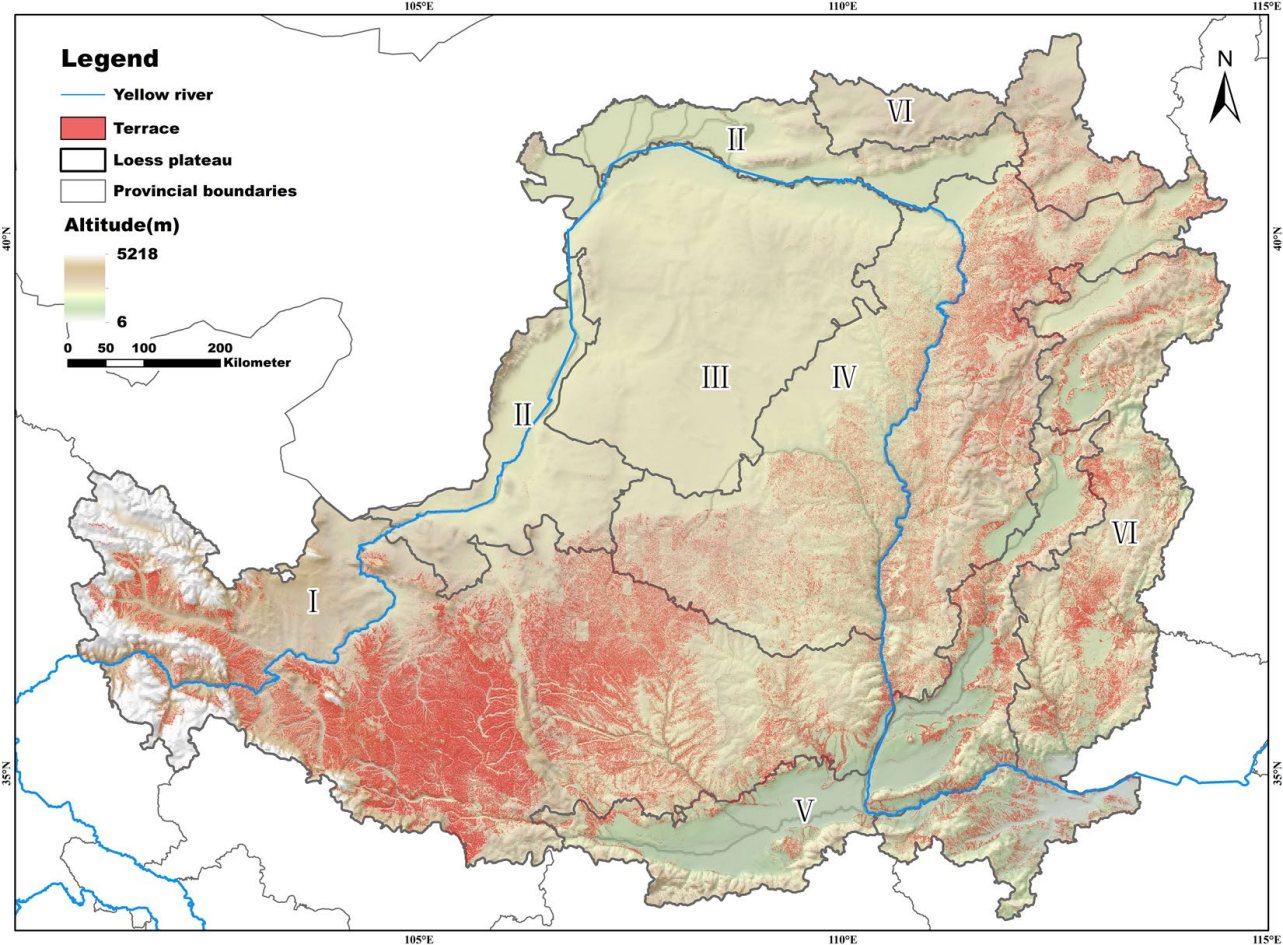
Map of terracing distribution results on the Loess Plateau(I: loess gully region; II: irrigation region; III: sand and desert region; IV: loess hilly and gully region; V: valley plain region; VI: earth-rocky mountain region).

We evaluated the accuracy of the spatial distribution map of terraces on the Loess Plateau (Fig. 4) using three approaches: (1) The accuracy of each step of model extraction was checked. (2) The accuracy of TDMLP was verified by region (pixel-by-pixel basis). (3) The actual usability and accuracy of TDMLP was verified in the field.

According to the type of test samples collected (see the training and validation data section) and the sample selection method, the terrace extraction accuracy of the model at each step of the pixel-by-pixel basis validation point evaluation was greater than 96% at the pixel scale (Table 1). The terrace extraction accuracy based on the UNet++ network model was 96.41%. Moreover, after correction based on topographic data, accuracy increased from 1.01% to 97.42% and from 0.45% to 97.84% after correction based on vegetation data. The final correction was carried out manually, resulting in an overall accuracy of 98.39%, which was deemed satisfactory.

**Table 1:** Results of a step-by-step verification of the accuracy of the Loess Plateau terrace distribution map (Kappa coefficient: 0.87).

Process	True class	Terrace	Non-Terrace	Users accuracy	Producer accuracy	Overall accuracy
	Predict class					
UNet++	Terrace	4807	387	92.55%	99.52%	96.41%
	Non-Terrace	23	6203	99.63%	94.13%	
DEM	Terrace	4922	272	94.76%	99.53%	97.42%
	Non-Terrace	23	6203	99.63%	95.80%	
GCL2020	Terrace	4974	220	95.76%	99.54%	97.87%
	Non-Terrace	23	6203	99.63%	96.57%	
Manual correction	Terrace	5033	161	96.90%	99.55%	98.39%
	Non-Terrace	23	6203	99.63%	97.47%	

The corrected data had a greater impact on the user accuracy improvement of the extracted terraces. As shown in Table 1, we found that although the user accuracy of the terrace map of the Loess Plateau based on the UNet++ network model had reached 92.55%, user accuracy was improved by 2.21%, 1%, and 1.14% after correction using topographic data, vegetation data, and manual correction, respectively. The end-user accuracy reached 96.90%, and the final kappa was 0.97, indicating good consistency. Using the above data analysis, we found that the topographic data had the most marked effect on enhancing the accuracy of mapping terraces on the Loess Plateau, followed by manual correction and vegetation data.

**Fig. 5:**
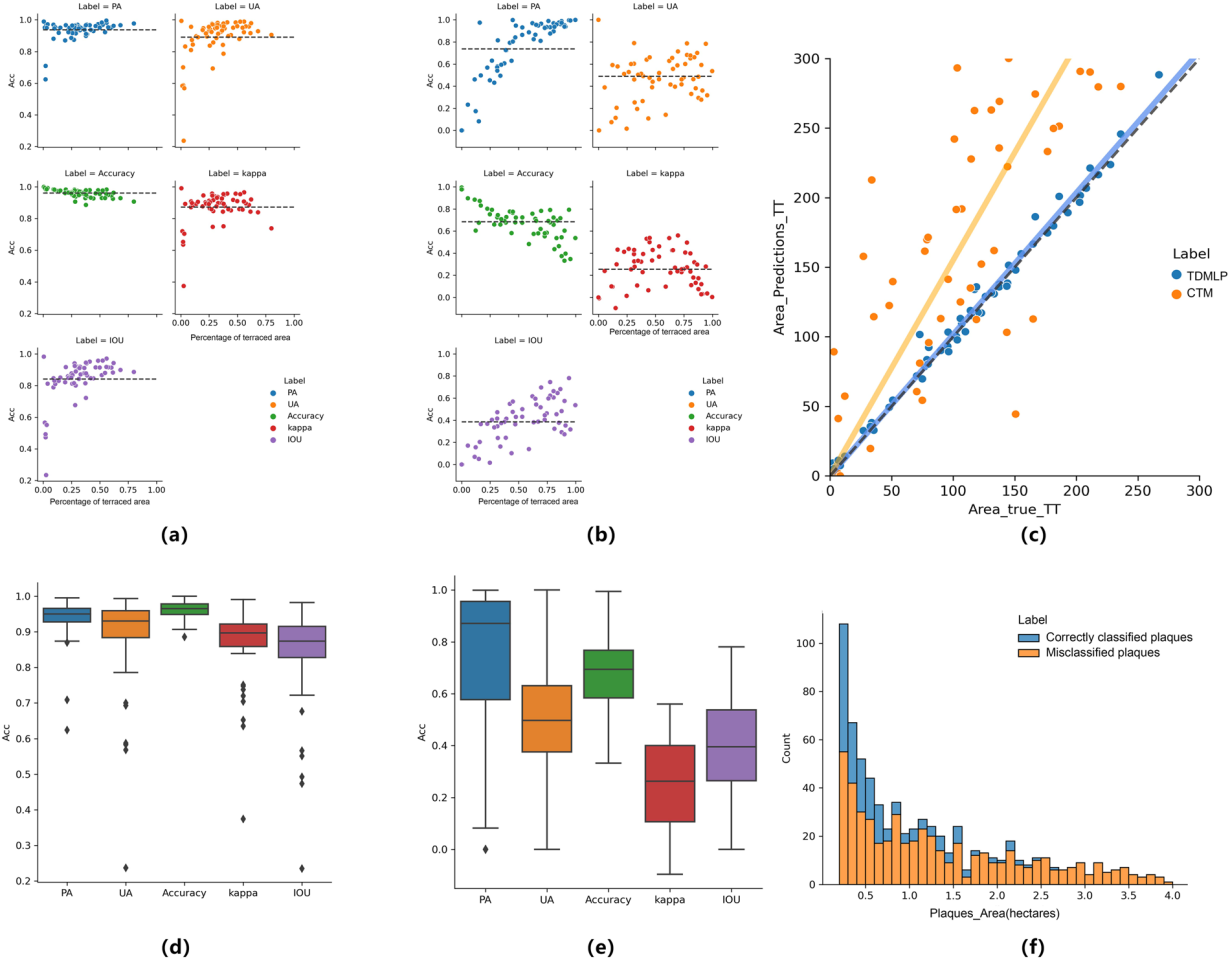
Accuracy validation results for TDMLP and CTM on the Loess Plateau, based on 60 squares containing terrace classification. (**a**) TDMLP accuracy on the Loess Plateau (including producer’s accuracy of terraces, user’s accuracy, total accuracy, kappa, IOU); (**b**) CTM accuracy on the Loess Plateau (including producer’s accuracy of terraces, user’s accuracy, total accuracy, kappa, IOU); (**c**) Comparison of the predicted and true values of area for TDMLP and CTM; (**d**) boxed maps of TDMLP at various accuracies; (**e**) boxed maps of CTM at various accuracies; (**f**) terrace patches in TDMLP sorted by patch area (blue is the number of mistaken patches; orange is the number of correct patches).

Due to the large area and complex and variable topography of the Loess Plateau, we divided the region into six major regions (loess hills and gullies, loess gullies, valley plains, earth-rocky mountains, irrigation, and sand and desert zones) to evaluate the accuracy of the results (Table 2). Tables 1, 2 show that the accuracy of each Loess Plateau region is above 98%. The accuracy of the sand and desert region and the valley plain region were both above 99% due to the use of vegetation correction data and manual correction, which greatly improved the accuracy of terrace mapping in the region. Relatively lower accuracy was achieved for the loess gully region, the loess hilly and gully region, earth-rocky mountainous region, and irrigation region. This was because the image had some non-terraced features with similar colour and texture in these areas, such as river floodplains and abandoned open-pit mines. Although the results were corrected for vegetation and topographic information, there were still some areas with errors due to the inherent errors of the corrected data. To better demonstrate our extraction results, we randomly intercepted some terraced areas in six districts and overlaid them with Google images of the area; the results are shown in Fig. 5. We found that the DLTEM method produced more effective terrace distribution results. First, according to the results shown in Fig. 5a,c,g), the proposed method is effective at distinguishing terraces from flat land, construction land, grassland, forest land, and other land types. Moreover, we found that the terrace edges extracted by this method are smoother, and the salt and pepper phenomenon in land cover extraction was effectively restrained, which improved the accuracy and reliability of the extraction results.

**Table 2:** Verification accuracy results for each partition of the Loess Plateau terrace distribution map.

Area	Correct	Mistake	accuracy
Loess gully region	4206	86	98.00%
Valley plain region	943	9	99.05%
Irrigation region	947	13	98.65%
Loess hilly and gully region	2037	41	98.03%
Sand and desert region	1212	3	99.75%
Earth-rock mountain region	1891	32	98.34%

From the field verification scale, we collected 815 sample points, the spatial distribution of which is shown in Fig. 4. Out of the total sample points, 700 points were terraced, and 115 points were non-terraced. The model identified 790 correctly extracted points and 25 incorrectly extracted points, indicating that the overall accuracy of the terraced distribution map products of the Loess Plateau was as high as 96.93%. This figure is slightly lower than the overall accuracy (97.74%) based on single-pixel sample point evaluation, but close to the terrace production accuracy of 96.42% determined by this evaluation method. This could be because the field validation route was mainly focused on the dense terraced areas, with fewer verifications of non-terraced points. The correct rate of field verification was close to that of single-pixel point verification and the accuracy is above 94% in all cases, indicating that the terrace distribution map of the Loess Plateau has high and credible accuracy.

In terms of the error analysis of the field verification points, we found that the error points were primarily concentrated in the northern part of the Loess Plateau (loess hills and gullies), that is, the northern part of Yulin and Yan’an cities in Shaanxi Province, China. The number of extracted error points was as high as 17. After comparing the images, we found that the source of error is primarily due to the different times at which the high-resolution satellite images were acquired. This resulted in large differences between the results of the image radiation correction and other images. Therefore, artificial adjustments were made to the area later. The distribution of other areas was more scattered, and the overall accuracy of the distribution pattern was the same as that of the per-pixel-based sample point evaluation method. The accuracy rate was higher in the regions with higher terrace density, and the regions with low terrace density were prone to errors.

### Comparison with other products

After searching for products on the terraces of the Loess Plateau, we found that only China Terrace Map (CTM)^12^ is currently available. Therefore, we compared DLTEM with CTM. We evaluated these two datasets in three ways and analysed the sources of uncertainty. In the first evaluation method, we compared the two data products in terms of spatial resolution, training dataset, extraction method, and accuracy evaluations (Table 3). In the second method, we compared the overall accuracy and patch-scale accuracy of the two products based on a spatial random squares validation method. In the third method, we compared the spatial homogeneity and differences between the two data products extracted in the Loess Plateau region (Fig. 5). Finally, we analysed the sources of CTM uncertainties and the advantages of TDMLP.

**Table 3:** Overall comparison of TDMLP and CTM, including spatial resolution, training dataset, extraction method, and accuracy evaluation (per-pixel-based validation).

	TDMLP	CTM
Data Source	High definition images	Landsat
Resolution	1.89	30
Training Data	7-county full area	Manual point selection
Extraction method	Deep Learning	Random Forest
Total number of validation points	11420	10875
Overall accuracy	98.39%	94.731%
Number of validation points	5194	1092
Producer precision	99.55%	79.945%
Number of user validation points	4830	1227
User accuracy	96.90%	71.15%
Kappa	0.97	0.72
IOU	0.96	0.60

The first method is based on the spatial comparison results of point validation. The spatial resolution of TDMLP is 1.89 m, whereas the spatial resolution of CTM is 30 m. The spatial resolution of TDMLP is much higher than that of CTM. Furthermore, the training data and extraction methods of both are distinct. CTM training data is mainly based on spatial manual point selection, with random forest as the primary classification method, whereas TDMLP training data is much larger, and a total of 7 counties in the Loess Plateau are selected. Deep learning was used as the classification method. In terms of accuracy evaluation, CTM selected 10,875 points at the national level to validate the product, whereas TDMLP selected 11,420 validation points at the Loess Plateau level, and selects more validation points in the Loess Plateau region compared to CTM. Therefore, the overall accuracy validation reliability of TDMLP in the Loess Plateau region is greater than that of CTM, and both TDMLP and CTM products focus on the accuracy of terrace types rather than non-terrace types. Therefore, we compared the user and producer accuracy of terrace type for both products. The validation results showed that the overall accuracy, user accuracy, producer accuracy, and kappa coefficient of TDMLP are 3.659%, 25.751%, 19.605% and 0.2465 higher than that of CTM. This indicates that TDMLP is more reliable than CTM in the Loess Plateau region and is outperforms CTM in terms of spatial resolution, reliability of accuracy validation, and accuracy validation results.

In the second step, we used a spatial random squares validation method to validate and compare the two products. We first applied the Icosahedral Snyder Equal Area Discrete Global Grid^36^ created by ArcGIS. The study area was divided into 79 regions (Fig. 5), and a randomly selected square with a size of 1,000*1,000 in each region was manually visually interpreted. The visual interpretation results were then overlaid with two products for validation. We examined validation using two scales: accuracy validation, and patch scale. For accuracy validation, we mainly used the method of Olofsson *et al*.^37^. At this step, we also added Intersection over Union (IOU)^38^ as one of the additional accuracy validation metrics since the two products are more concerned with the accuracy of terrace type classification. Validation includes user accuracy, producer accuracy, total accuracy, kappa coefficient, and IOU. Aside from users concerned with the accuracy validation of the products, terrace size and terrace fragmentation are equally important. The number of terrace patches and the average patch area size are essential indicators of terrace fragmentation and one of the critical aspects of product accuracy. Therefore, we will use three indicators from the terrace area, the number of terrace patches, and the average area of terrace patches as the validation on the product patches level. In the third step, because we were more concerned with the classification of terraces, we selected 60 squares with terraces from the 79 squares for validation.

**Fig. 6:**
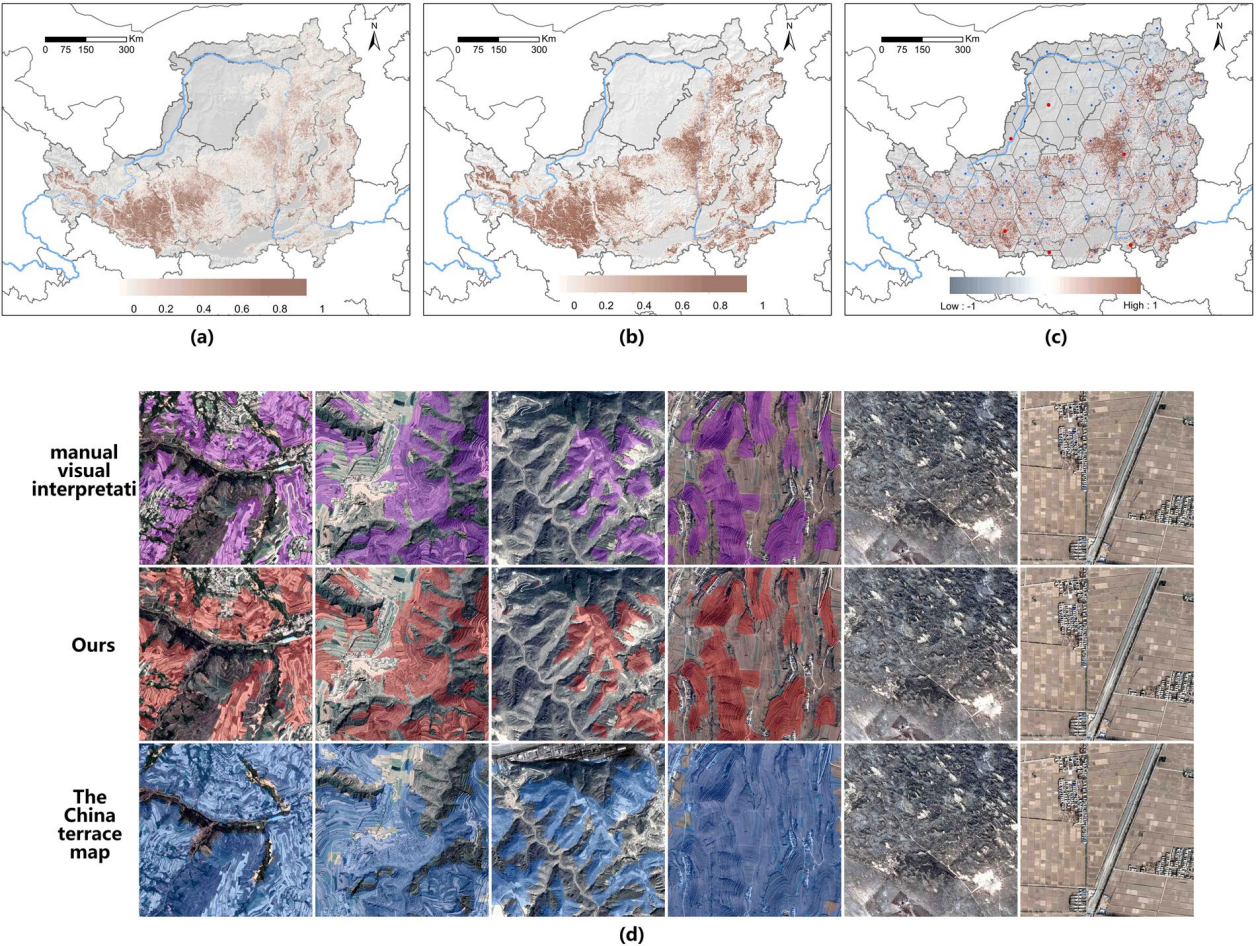
The results of TDMLP and CTM on the Loess Plateau are shown with resampling to 1 km*1 km of terraces percentage. (**a**) Classification results of TDMLP on the Loess Plateau; (**b**) Classification results of CTM on the Loess Plateau. (**c**) Overlay results of TDMLP and CTM (the proportion of terraces of TDMLP minus the proportion of terraces of CTM, darker brown means more terraces predicted by CTM than those predicted by TDMLP, darker blue means more terraces predicted by TDMLP than those predicted by CTM); (**d**) Visual interpretation, TDMLP extraction results, and CTM extraction results of the case areas in the five of the Loess Plateau extraction results and high-resolution satellite image overlay results (the location of the overlay case area is the area marked by the red original edge in figure(c)).

The validation results are as follows. In terms of accuracy validation, we found that the mean user accuracy, producer accuracy, and total accuracy distribution of TDMLP were 0.8903 (S^2^ = 0.1287), 0.9365 (S^2^ = 0.0576), 0.9606 (S^2^ = 0.0234) (Fig. 6). The results of CTM in the Loess Plateau were 0.4902, 0.7371, and 0.6840. The accuracy comparison results showed that the user accuracy, producer accuracy, and total accuracy of TDMLP were all more than 20% higher than those of CTM products, with the user accuracy being 40% higher. In the kappa consistency test, TDMLP had a consistency of 0.8719 (S^2^ = 0.0976), whereas CTM had a consistency of only 0.2555, a difference of 61.64%. Therefore, the consistency of TDMLP product results with actual classification results was high, whereas the consistency of CTM was low, and there was a large error. Finally, we characterised the accuracy of terrace classification using IOU, and the IOU of TDMLP was 0.8413 (S^2^ = 0.1315), whereas that of CTM was only 0.3852. The difference between them was as high as 45.61%. This indicates, on the one hand, that there is considerable uncertainty in the use of CTM on the Loess Plateau, and that meeting the accuracy requirements in many terrace studies is challenging. On the other hand, all TDMLP accuracy assessments were greater than 89%, and the kappa coefficient was also 0.87, indicating strong consistency, and the IOU of our terrace types of interest was above 84% with high accuracy. It can effectively meet the requirements of future terrace-related studies, such as those on the prevention of soil erosion, slowing down land degradation, improving production capacity, and ecological protection.

To predict the terraced area, we superimposed the two products with the actual results of manual visual interpretation of 60 areas. We found that TDMLP had an R² of 0.9969 and a prediction area error of 1.0176 ± 0.0147 (P < 0.01). In contrast, CTM had an R² of 0.8716 and a predicted area error of 1.5501 ± 0.155 (P < 0.01). Although both TDMLP and CTM overestimated the terraced area of the Loess Plateau, TDMLP was closer to the true value and only overestimated it by 1.76% ± 1.47%. In contrast, CTM overestimated the terraced area of the Loess Plateau by 55.01% ± 15.5%. Therefore, CTM is fraught with uncertainty, making it difficult to accurately estimate the scale of terraces on the Loess Plateau. In contrast, TDMLP had a lower error and can effectively estimate the scale of terraces on the Loess Plateau.

A total of 839 terrace patches were extracted by manual visual interpretation. TDMLP covered 824 patches, with 2% of them omitted. We then compared the actual number of patches to the number of patches provided by the product, and found that TDMLP overestimated the number of patches by 12.45% (R² = 0.708). The CTM covered 692 patches, and 17.52% of the patches were omitted. The CTM predicted 358 patches, underestimating the number of patches by 57.33%. This indicates that the CTM product is difficult to study at the patch scale, while the TDMLP product has a clear advantage at the terrace patch scale.

We also counted the correct and incorrect patches by area, and the statistical results are as follows. We found that as the area of terrace patches increases, so does the percentage of correct terrace patches. Furthermore, the misclassified terrace patches were mainly concentrated in the terrace patches below 0.7 ha (Fig. 6), accounting for 68.5% of the total misclassified patches. Because terrace patches smaller than 0.7 ha account for less than 3% of the total terrace area, this product can effectively support the study of terraces at the patch scale (e.g., terrace fragmentation).

From the average area of patches, we found that the average size of patches predicted in TDMLP was 93.43% of the total average area of patches (R² = 0.7228). This indicates that the predicted average patch area of terraces in TDMLP was underestimated by 6.57% compared to the whole terrace patch area. In contrast, the average area of patches predicted in the CTM was 3.6 times larger than the true average area of patches (R² = 0.2338). This indicates that the predicted average terrace patch area in the CTM is overestimated by 2.6 times more than the true terrace patches. Thus, TDMLP outperformed CTM in both average patch size of terraces.

In the third step, we compared the spatial distribution and differences of the two products in the Loess Plateau region. To better compare the two products, we resampled the results to a spatial resolution of 1 km as a percentage of terraces. First, we found that the spatial distribution of the two terrace products was more similar (Fig. 5a,b). The terraces are mainly distributed in the plateau gully area, loess hills and gully area, earth and stone mountain area, and part of the river valley plain area. Furthermore, the scale of terraces in the plateau gully area is the greatest, as is the density of terraces distribution. This indicates that the two products are regionally consistent in spatial distribution. Second, we found that the local spatial distribution of the two terrace products was more different (Fig. 5c). The value of each raster represents the difference in terrace content. Figure 5 shows that the parts with larger differences are mainly concentrated in the Loess hilly and gully region and the Loess gully region, where the terraces of CTM are generally larger in size than TDMLP. According to the d in Fig. 5d, the misclassification and mixed image pixels of the terraces in CTM are more severe. In contrast, the edges of TDMLP are smoother than those of CTM, with less misclassification and less influence of mixed pixels. Therefore, the terraces of CTM were larger in scale due to terrace misclassification and mixed pixels at the edges of terrace patches. In summary, TDMLP can provide spatial distribution products of terraces with higher accuracy and spatial resolution compared with CTM.

In terms of error classification, CTM errors are primarily caused by three factors: mask data, mixed pixel, and classification methods. First, CTM mask data has low accuracy in mountainous areas. CTM uses the 2010 GlobeLand30 cropland data as a mask. Although this data is widely used globally and has achieved good results, there are some limitations to using this map as mask data to identify terraces. (1) Spatial heterogeneity is one of the major factors affecting the accuracy of land products. Although the correct rate of GlobeLand30 cropland data is as high as 79.16%^39^, the accuracy of GlobeLand30 cropland data in mountainous areas is much lower than in plain areas due to higher fragmentation of cropland patches and spatial heterogeneity in mountainous areas. Chen X^40^. used 506 validation samples to verify the accuracy of GlobeLand30 in cultivated land in Shaanxi province and found that the accuracy was over 80% in the plain ecological zone and only 60% in the desert and semi-desert and forest-steppe ecological zones. The difference between the two is close to 20%. (2) There is a temporal error between the mask data and the CTM product results. CTM is extracted from a spatial distribution map of Chinese terraces in 2018, but the cropland data of GlobeLand30 is from 2007 to 2010. In recent years, the outline of the comprehensive management plan for the Loess Plateau area (2010–2030) has been promulgated, with a planned area of 2.608 million hectares for slope-to-terrace conversion, making it one of the core areas of slope-to-terrace conversion projects in the country. Hence, the CTM has an error of nearly 10 years from the base map used, increasing the uncertainty and reducing the accuracy of the CTM. The base map used by TDMLP is mainly based on 2018, which effectively reduces the influence of time on terrace extraction.

Furthermore, the mixed pixel of CTM is serious with a CTM resolution of 30 m, which greatly affects the accuracy of CTM and the estimation of terrace areas in China. The mixed pixels introduce considerable uncertainty into pixel-level remote sensing image classification, which is the primary limitation of the accuracy and area measurement accuracy of remote sensing producer. Moreover, the fine fragmentation of target features is an important factor in amplifying the influence of mixed pixel. CTM has a resolution of 30 m, and 12 grids are equal to 1 hectare. Terraces as one of the important types of arable land in mountainous areas. It is more restricted by topography and the terrace patches are more trivial, so the 30 m spatial resolution can hardly meet the needs of actual terrace extraction. While the spatial resolution of TDMLP is 1.89 m, which can effectively reduce the influence of mixed pixel and thus improve the accuracy.

Third, although classic random forests have been widely demonstrated to have high accuracy and efficiency in remote sensing classification, most random forest application studies use target pixels as training targets, and less consider the neighbourhood information of target pixels or consider only a small range of neighbourhood information^41^ (for example, 3*3 or 5*5), so there is a limitation for feature extraction with rich morphological structure features. In contrast, deep learning techniques have considerable advantages in acquiring semantic information about target objects and actively extracting high-level features of target objects. Convolutional neural networks (CNN)^42^ are currently used in computer vision applications. This network tends to capture a broader range of target pixel neighbourhood information (for example, 256*256). Therefore, deep learning techniques have clear advantages when it comes to extracting features with distinct morphological structures. Terraces are primarily defined based on their morphological structural features. Hence, deep learning outperforms classical random forest for terrace classification.

Although CTM maps the spatial distribution of terraces on the Loess Plateau for the first time, its spatial resolution and classification accuracy are low due to limitations in its mask data, mixed pixel, and classification methods. This causes not only serious errors in describing the spatial distribution of terraces and estimating the scale of terraces, but also makes the current classification accuracy difficult to meet further terrace-related research on the Loess Plateau.

TDMLP can not only provide more practical information on the spatial distribution of terraces and more accurate estimation of terrace scales, but it can also enable the study of terraces at the patch scale. It can also provide effective data support for future research on the effects of terraces on soil conservation, slowing land degradation, improving economic income, reducing environmental pollution on the Loess Plateau, reducing data errors, and improving the scientific validity and credibility of conclusions.

### Uncertainty analysis

Although the DLTEM has a high accuracy for the regional high-resolution distribution map of terraces in the Loess Plateau, there are still some uncertainties in some areas. The main sources of uncertainty are training samples, image quality, and correction data.

First, in terms of the training samples, due to the complex and heterogeneous landscape of the Loess Plateau, it is difficult to include all of the characteristics (colour, morphology, texture) and features of the terraces when selecting training samples, which may lead to the misclassification of terraces types in some regions because they are not in the training samples.

Second, there are two main factors to consider in terms of image quality. On the one hand, the high-resolution images are not obtained simultaneously, and there is extensive splicing in some areas. Since spliced images have seasonal characteristics, terraces are missed and misidentified in some areas. On the other hand, due to the difficulty of acquiring images in some areas of the Loess Plateau and the low quality of the acquired images, the textural and morphological features of the terraces are not distinct, resulting in the occurrence of missed terrace scores.

Finally, in terms of data source correction, although the result can effectively remove the error caused by similar spectral anomalies after the correction of vegetation and terrain information, the error still occurs in some areas due to the error inherent in the correction data sources.

## Usage Notes

This study was a novel attempt to extract terraces at a regional scale using an image recognition method in deep learning. The framework model of the study was a UNet++ deep learning network, rather than a traditional land type classification. Based on the typical characteristics of terraces, we constructed a deep learning-based terrace extraction model and mapped the spatial distribution of terraces on the Loess Plateau with a spatial resolution of 1.89 m for the first time. Furthermore, the total area of terraces on the Loess Plateau was calculated using the error matrix model-assisted estimation method. The total area is 9.02 ± 0.32 million hectares.

For the first time, the spatial distribution of meter-scale terraces in the region has been mapped, which is crucial for future assessment of the contribution of terraces to food security, soil and water conservation, carbon cycling, landscape ecological values, and guiding the Chinese government to make precise poverty alleviation and rural revitalization decisions. Many studies have found that terraces are an effective measure for soil and water conservation on the Loess Plateau, and their contribution to soil and water conservation is one of the key factors in assessing whether new terraces should be built in this region. However, these studies have focused only on a few small watersheds. Evaluations for the entire Loess Plateau region have not yet been performed. Therefore, we can apply the high-resolution terrace distribution map in conjunction with soil, precipitation, temperature, and slope data to estimate the reduction in soil erosion on terraces on the Loess Plateau using generalized erosion equations. Terraces are an important arable land resource on the Loess Plateau, and it is crucial to understand their current utilization for local sustainable development. Furthermore, we can use high-resolution terrace distribution maps overlaid with time-series spectral information to study planting structure, terrace abandonment, and other characteristics of terraces on the Loess Plateau in detail.

## Data Availability

The source code used the Python language. The source code contains five sections: data_loader5_shanxitezhengqu_LP.py, unet_2d.py, data_preprocess.py, train_shanxitezhengqu_LP.py, Config_shanxitezhengqu_LP.py. The source code can be downloaded at https://github.com/LYHTTUCAS1/code.
